# Potential Therapeutic Effect and Mechanisms of Mesenchymal Stem Cells-Extracellular Vesicles in Renal Fibrosis

**DOI:** 10.3389/fcell.2022.824752

**Published:** 2022-03-11

**Authors:** Chunling Liao, Guangyong Chen, Qian Yang, Yiping Liu, Tianbiao Zhou

**Affiliations:** Department of Nephrology, The Second Affiliated Hospital of Shantou University Medical College, Shantou, China

**Keywords:** chronic kidney disease, renal fibrosis, mesenchymal stromal/stem cells, extracellular vesicles, acute kidney injury

## Abstract

Renal fibrosis (RF) is central pathological pathway for kidney diseases, with the main pathological features being the aberrant accumulation of myofibroblasts that produce accumulation of extracellular matrix in the renal interstitium and glomeruli. Acute kidney injury (AKI) and chronic kidney disease (CKD) are associated with RF. Current treatment strategies for RF are ineffective. Mesenchymal stem cells (MSCs) have been found to be able to treat organ fibrosis including RF, but they have some safety problems, such as cell rejection, carcinogenicity, and virus contamination, which limit the application of MSCs. However, current studies have found that MSCs may exert their therapeutic effect by releasing extracellular vesicles (EVs). MSC-EVs can transfer functional proteins and genetic material directly to the recipient cells. As non-cell membrane structures, MSC-EVs have the advantages of low immunogenicity, easy preservation, and artificial modification, but do not have the characteristics of self-replication and ectopic differentiation. Therefore, EVs are safer than MSCs for treatment, but might be less effective than MSCs. Recent studies have also found that MSC-EVs can improve renal function and pathological changes of RF. Thus, this review summarizes the therapeutic effect of MSC-EVs on RF and the mechanisms that have been discovered so far, so as to provide a theoretical basis for the further study of the role of MSC-EVs in treating RF diseases.

## Introduction

Renal fibrosis is the common pathological pathway for kidney diseases, and the main pathological features are the aberrant accumulation of myofibroblasts that produce accumulation of extracellular matrix in the renal interstitium and glomeruli. Acute kidney injury (AKI) and chronic kidney disease (CKD) are associated with RF diseases. CKD is one of the most common diseases that endanger human health and life, affecting more than 10% of the world’s population (Djudjaj and Boor, 2019). It is estimated that by 2030, 14 per 100,000 people may die from CKD (Xu et al., 2021). While CKD progresses to the terminal stage, renal fibrosis (RF) is the final pathological manifestation of CKD (Xu et al., 2021). Furthermore, AKI is also associated with RF. When AKI is not corrected in time, it develops into RF. However, most cases of RF result from CKD. RF is mainly characterized by excessive deposition of extracellular matrix, which destroys the renal structure, impairs function, and leads to organ failure (Djudjaj and Boor, 2019). According to the histological structure, RF can be divided into 4 parts: glomerular sclerosis (GS), renal interstitial fibrosis (RIF), arteriosclerosis, and perivascular fibrosis (APF) (Xu et al., 2021). RF has become the ultimate target for the treatment of CKD. Currently, clinically available drugs and therapeutic measures include angiotensin-converting enzyme inhibition, angiotensin receptor blocker, optimal blood pressure control, and sodium bicarbonate for metabolic acidosis, mainly in order to delay the progression of CKD and to prevent CKD-related complications (Humphreys, 2018). Despite these treatments, the outcome of CKD is still poor. Therefore, it is very important to find a targeted drug or treatment strategy that can effectively prevent RF. However, current treatment strategies for RF are ineffective.

At present, a large number of studies have found that mesenchymal stem cells (MSC) have a therapeutic effect on organ fibrosis, such as liver fibrosis (Alfaifi et al., 2018), lung fibrosis (Chen et al., 2018; Chuang et al., 2018; He et al., 2018), kidney fibrosis (Zhuang et al., 2019), and heart fibrosis (Li et al., 2015). MSCs are mainly derived from bone marrow, adipose tissue, umbilical cord and other perinatal tissues, such as the amniotic membrane, chorionic membrane, placental decidua, and Wharton colloid (Alfaifi et al., 2018; Farge et al., 2021), and have the characteristics of adhesion to plastic with a fibroblast-like morphology; expression of CD73, CD90, and CD105 (>95%MSC); lack expression of hematopoietic and endothelial markers (such as CD45, CD34, CD14, CD11b, and human leukocyte antigens (HLA) in humans); and have the ability to differentiate into osteoblasts, adipocytes, and chondroblasts *in vitro* (Lelek and Zuba-Surma, 2020; Farge et al., 2021). They have pro-angiogenic, immunosuppressive, and anti-fibrotic properties (Farge et al., 2021), inhibit inflammation, and promote tissue repair (Shi et al., 2018). In addition, MSCs are easy to harvest, have low immunogenicity and can be expanded *in vitro*, all characteristics of a cell population capable of treating organ fibrosis (Li et al., 2021). However, MSC therapy requires cell transplantation, which could possess safety problems (Zhang et al., 2020; Racchetti and Meldolesi, 2021), such as cell rejection, unexpected immune responses, toxicity, carcinogenicity, and viral contamination, as well as the need for cell transportation and storage before transplantation (Lelek and Zuba-Surma, 2020), which therefore limit the application of MSCs.

An increasing number of studies have indicated that MSCs may play a therapeutic role by releasing extracellular vesicles (EVs). MSC-EVs are round vesicles surrounded by a phospholipid bilayer, and carry lipids, proteins, and nucleic acids (Lelek and Zuba-Surma, 2020). They can be divided into the different subclasses according to different characteristics: 1) biological origin: endosome-originated “exosomes”, plasma membrane-derived “ectosomes” (microparticles/microvesicles); 2) size: “small EVs” (sEVs) and “medium/large EVs” (m/lEVs), with defined ranges, for instance, <100 nm or <200 nm [small], or >200 nm [large and/or medium]; 3) density: low, middle, high, with each range defined; 4) conditions or cell of origin: podocyte EVs, hypoxic EVs, large oncosomes, and apoptotic bodies. Currently, they can be separated and concentrated mainly by differential ultracentrifugation or other techniques, such as density gradients, precipitation, filtration, size exclusion chromatography, and immuno-isolation. In addition, for better specificity of EVs or EV subtype separation, one or more additional techniques can be used following the primary step, such as washing in EV-free buffer, ultrafiltration, application of density gradients (velocity or flotation), or chromatography, However, it is currently still challenging to isolate specific EV subtypes at defined purity and more new techniques are needed (Théry et al., 2018).

MSC-EVs are able to transfer functional proteins and genetic material directly to recipient cells, achieving the same therapeutic effect as MSCs (Wang et al., 2020a; Thalakiriyawa et al., 2021). On the other hand, compared with MSCs, MSC-EVs have lower immunogenicity, easy preservation, and the potential for being artificially modified (Li et al., 2021), and lack potentially dangerous properties, such as self-replication, ectopic differentiation, tumor formation, and genetic instability (Jafarinia et al., 2020). Therefore, MSC-EVs can replace MSCs in the treatment of inflammation and immune-related diseases (Wang et al., 2020a), such as organ fibrosis (Huang and Yang, 2021). This review was conducted to describe the role of MSC-EVs in the treatment of RF, including MSC-EVs from different sources to different animal models, and summarize the potential biological mechanisms involved.

### MSC-EVs in the Treatment of RF

1) Effects of MSC-EVs in mice or rats with AKI after ischemia-reperfusion:

Deterioration of renal function is an important clinical feature of AKI. If the injury is not corrected in time, AKI will develop into RF. Zou et al. (2016a) found that EVs from human umbilical cord-derived mesenchymal stromal cells (HUMSCs) could reduce the levels of serum creatinine (Cr) and blood urea nitrogen (BUN), and alleviate the necrosis and dilation of renal tubules. Zou et al. (2016b) showed that in addition to lowering the BUN and Cr, HUMSC-EVs also could reduce apoptosis and promote the proliferation of renal cells, increase the capillary density in renal tissue, and ultimately relieve RF. Cao et al. (2020) found that EVs from human placenta derived MSCs (HP-MSC-EVs) could inhibit the elevation of BUN and Cr, and greatly reduce the formation of tubular protein casts and necrotic areas in proximal renal tubules. In addition, HP-MSC-EVs could reduce the expression of kidney injury molecule-1 (Kim-1), a specific and sensitive biomarker of renal tubular injury, in proximal renal tubules. Liu et al. (2020) injected HP-MSC-EVs into the renal cortex, and found that they could reduce BUN, Cr, and the formation of necrotic tubules, hyaline casts and the fibrotic area, demonstrating HP-MSC-EVs can have a huge anti-fibrotic effect in AKI. Zhang et al. (2020) found that renal injection of HP-MSC-EVS could reduce BUN, Cr and Kim-1, and alleviate RF. Zhang et al. (2016) found that EVs derived from human Wharton’s jelly mesenchymal stromal cells (HWJMSC-EVs) could decrease BUN, Cr and neutrophil gelatinase-associated lipocalin (NGAL) as a marker of kidney damage. It also could reduce the number of apoptotic cells and relieve the damage of renal tubules. Du et al. (2021) found that HWJMSC-MVs could lead to a decrease in Cr and BUN, and could also reduce the renal content of procollagen III (a fibrosis marker) and relieve renal tubular epithelial cell degeneration/necrosis and interstitial inflammatory cell infiltration. Gu (2016) found that HWJMSC-MVs could reduce not only serum BUN and Cr but also apoptosis of the renal tubular epithelium. Zhao et al. (2021) also found that EVs from mouse bone marrow-derived mesenchymal stem cells (BM-MSCs) could significantly reduce BUN and Cr, and reverse the rate of increase of renal tubular necrosis, Kim-1 and apoptosis, and reduce the infiltration of macrophages in renal tissues. Therefore, MSC-EVs from different cell sources could improve renal function and pathological injury in mice or rats with AKI after ischemia-reperfusion, thus inhibiting or delaying the development of RF.

2) Effects of MSC-EVs in rat models of unilateral ureteral obstruction (UUO) or stricture:

The UUO model can simulate the characteristics of chronic RF. Wang et al. (2020b) found that in UUO model, BM-MSC-EVs from young rats could significantly reduce blood BUN, Cr, and uric acid (UA). The MSC-EVs also decreased deposition of extracellular matrix in renal tissue and improved renal tubule dilation, apoptosis, and necrosis, as well as the appearance of proteinaceous casts in the tubules. Luo et al. (2018) found in the rat model of ureteral stenosis that MSC-EVs administered *via* renal artery could significantly improve ureteral fibrosis, restore ureteral morphological development, improve hydronephrosis, reduce renal dysfunction, and relieve transformational growth factor -β 1 (TGF-β 1)-induced fibrosis. Therefore, MSC-EVs can improve renal function and RF in rats with renal injury induced by UUO or ureteral stenosis.

3) Effects of MSC-EVs in rat or mouse models with renal injury induced by drugs:

Toxic and side effects of certain drugs can result in kidney fibrosis. Ramírez-Bajo et al. (2020) found that BM-MSC-EVs reduced blood BUN level and increased survival in mice with chronic cyclosporine nephrotoxicity (75 mg/kg cyclosporine peritoneally injected for 4 weeks). BM-MSC-EVs can also reduce the formation of renal tubular vacuolation, casts and cysts, thus improving histological damage. Kholia et al. (2020) found that in a mouse model of aristolochic acid nephropathy (AAN) (celiac injection of 4 mg/kg aristolochic acid, once a week for 4 weeks), intravenous BM-MSC-EVs could reduce blood BUN and Cr. BM-MSC-EVs also reduced the infiltration of CD45-positive immune cells, fibroblasts, and pericytes into the interstitial tissue after injury, and relieved the renal tubular necrosis as well as interstitial fibrosis. Grange et al. (2019) found that, in a mouse model with streptozotocin (STZ)-induced diabetic nephropathy (abdominal injection of 37 mg/kg STZ for 4 consecutive days), human BM-MSC-EVs treatment could improve the functional parameters of diabetic mice, such as the albumin/creatinine excretion rate, blood BUN and blood Cr; and could also inhibit RF. Zhong et al. (2018) established a diabetic nephropathy mouse model, where serious pathological changes included vacuole and particle denaturation to a flattened even flaky or diffuse atrophy in renal tubular epithelial cells, gradual expansion in the lumen, gradual increases in protein casts, and mononuclear cell infiltration to focal and even multifocal fibrosis in renal interstitial tissue. In this model, HUMSC-EVs injected into the tail vein could decrease BUN as well as Cr levels, and reverse the above-mentioned renal pathological manifestations. Therefore, MSC-EVs relieve renal dysfunction and RF in rat and mouse models of drug-induced renal injury. In conclusion, in different animal models of renal injury, MSC-EVs from different sources can significantly improve renal function, promote renal tubule recovery, and improve the pathological manifestations of RF.

### Potential Mechanisms by Which MSC-EVs Improve RF *in vivo* and *in vitro*

**FIGURE 1:**
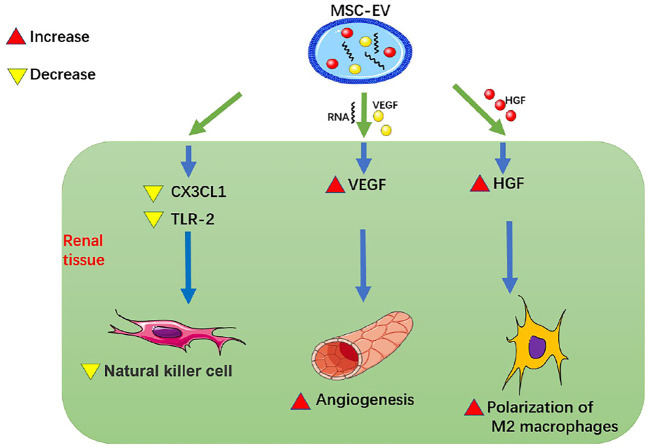
MSC-EVs promote renal angiogenesis and reduce renal inflammatory cell infiltration. MSC-EV, mesenchymal stem cell-extracellular vesicle; VEGF, vascular endothelial-derived growth factor; HGF, hepatocyte growth factor; CX3CL1, C-X3-C motif chemokine ligand 1; TLR-2, Toll-like receptor 2.

1) At the organ level, MSC-EVs promote renal angiogenesis and reduce renal inflammatory cell infiltration (Figure 1; Table 1):

**TABLE 1:** Summary of potential therapeutic effect and mechanisms of mesenchymal stem cells-extracellular vesicles in renal fibrosis.

Author, year	Models	MSC-EVs	MicroRNAs/DNAs in EV	Proteins in EV	Reactions in cells or kidney
Zhang 2020 (Zhang et al., 2020)	IRI (mouse); hypoxia/reoxygenation-induced injury (HK2)	HP-MSC-EVs	miRNA let-7a-5p	一	1.miRNA let-7a-5p↑ → CASP3↓→ renal cell apoptosis↓; 2.miRNA let-7a-5p↑ → RragD↓→ p-mTOR/p62↓, the ratio of LC3-II/LC3-I↑→ renal injury↓
Zou 2016 (Zou et al., 2016a)	IRI (rat)	HUMSC-EVs	miRNAs	一	CX3CL1/TLR-2↓ → NK cell infiltration↓ → renal inflammation↓→ renal injury ↓
Zou 2016 (Zou et al., 2016b)	IRI (rat); hypoxia-injured NRK-52E cells	HUMSC-EVs	一	VEGF	VEGF↑ → angiogenesis↑ → renal injury↓
Cao 2020 (Cao et al., 2020)	IRI (mouse)	HP-MSCs-EVs	microRNA-200a-3p	一	1.microRNA-200a-3p↑ → Nrf2/SOD2↑, Keap1↓→ Keap1-Nrf2 signaling pathway activation → mitochondrial antioxidant defense↑(ATP production/mtDNA↑, mitochondrial fragmentation↓) → TEC oxidative damage ↓→ renal injury↓ 2. TNF-α/caspase-8 ↓→ inflammation/cell apoptosis↓→ renal injury↓
Liu 2020 (Liu et al., 2020)	IRI (mouse)	HP-MSC-EVs	一	一	GRP78/CHOP/cleaved caspase-12↓→ endoplasmic reticulum stress↓
Zhang 2016 (Zhang et al., 2016)	Right nephrectomy-left renal ischemia induced AKI (rat); hypoxia-injured NRK-52E cells	HWJMSC-EVs	一	一	Nrf2/ARE activity in the nucleus↑ → HO-1/SOD↑ →renal tubular injury↓
Du 2021 (Du et al., 2021)	Ischemia-partial nephrectomy induced AKI (rat); LPS-stimulated THP-1 macrophages model	HWJMSC-MVs	一	HGF	HGF↑ → polarization of M2 macrophages↑ → renal fibrosis↓
Gu 2016 (Gu, 2016)	IRI (rat); ATP depletion-induced IRI (NRK-52E cells)	HWJMSC-EVs	miR-30b/c/d	一	miR-30b/c/d ↑ → mitochondrial DRP1↓→apoptosis/renal injury↓
Zhao 2021 (Zhao et al., 2021)	H_2_O_2_-induced oxidative injury (HK-2)	HUMSC-EVs	TFAM mRNA/mtDNA	一	TFAM mRNA↑ → TFAM protein↑ → stability of the TFAM-mtDNA complex↑ → mtDNA ↑ → mitochondrial OXPHOS↑
Wang 2020 (Wang et al., 2020b)	TGF-β1-induced EMT (HK-2 cells)	Rat BM-MSC-EVs	miR-294/miR-133	一	miR-294/miR-133 ↑ → phosphorylation of SMAD 2/3 and ERK1/2↓→α-SMA↓E-cadherin↑→ EMT↓
Luo 2018 (Luo et al., 2018)	US (rat)	Rat BM-MSC-EVs	miR-29b-3p/miR-19b-3p/miR-130a-3p/miR-590-5p/miR-146a-5p/miR-181a-5p	一	TGF-β1 ↓, Smad3 ↓→Col I ↓, Fib↓→
Ramírez-Bajo 2020 (Ramírez-Bajo et al., 2020)	Cyclosporine-induced chronic renal injury (mouse)	Mouse BM-MSC-EVs	一	一	PAI1/TIMP-1/IFN-γ↓→ EMT/renal fibrosis↓
Kholia 2020 (Kholia et al., 2020)	Aristolochic acid -induced renal injury (mouse)	Human BM-MSC-EVs	hsa-miR-194-5p/hsa-miR-192-5p/mmu-miR-378a-3p	一	hsa-miR-21-5p/hsa-miR-34a-5p/hsa-miR-34c-5p/hsa-miR-132-3p/hsa-miR-342-3p/mmu-miR-212-3p/hsa-miR-214-3p↓, hsa-miR-194-5p/hsa-miR-192-5p/mmu-miR-378a-3p↑→ LTBP1/α-SMA/TGF-β1/Col1a1↓→ renal injury↓
Grange 2019 (Grange et al., 2019)	STZ-induced DN (mice)	Human BM-MSCs-EVs	hsa-miR-302c-3p/hsa-let-7b-5p/hsa-miR-1243/hsa-miR-100-5p/hsa-let-7e-5p/hsa-miR-125b-5p/hsa-miR-21-5p/hsa-miR-30a-5p	一	hsa-miR-302c-3p/hsa-let-7b-5p/hsa-miR-1243/hsa-miR-100-5p/hsa-let-7e-5p/hsa-miR-125b-5p/hsa-miR-21-5p/hsa-miR-30a-5p↑ → TGF-β/α-SMA/collagen I↓→renal fibrosis↓
Zhong 2018 (Zhong et al., 2018)	hyperglycemia and hyperuricemia-induced injury (HK-2 cells); the diabetic nephropathy model with hyper uric acid (mice)	HUMSCs-MVs	miR-451a	一	miR-451a↑ → P15/P19 (cell cycle inhibitors)↓→α-SMA↓E-cadherin↑→ EMT↓
Zou 2014 (Zou, 2014)	IRI (rat)	HWJMSC-MVs	miR-16/miR15b	一	CX3CL1 ↓ → macrophages infiltration↓ → renal inflammation↓ →renal fibrosis↓
			/miR15a		
Lindoso 2014 (Lindoso et al., 2014)	ATP depletion-induced IRI (HK2)	Human BM-MSC-EVs	In EVs: miR-410/miR-495/miR-548c-5p; miR-485-3p/miR-let-7a	Not in EVs: miR-375/miR-548c-5p/miR-561	1. miR-375/miR-548c-5p/miR-561↑ → SHC1↓ → reactive oxygen species↓→ mitochondrial depolarization↓ → mitochondrial dysfunction↓; 2.miR-410/miR-485-3p/miR-548c-5p/miR-561↑ →Smad3/Smad4↓→ collagen↓→EMT/renal fibrosis↓ 3. miR-410/miR-495/miR-548c-5p/miR-let-7a↑→ CASP3↓→ cell apoptosis↓; 4.miR-375/miR-495/miR-548c-5p↑→ CASP7↓→ cell apoptosis↓
Cao 2021 (Cao et al., 2021)	TGF-β1-induced injury (HK2 cells); UUO (mouse)	MSC-EVs	miR-133b	一	miR-133b↑ → CTGF↓→ α-SMA/FN/collagen 3A1↓, E-cadherin↑→ EMT/renal fibrosis↓

Note: IRI, ischemia-reperfusion injury; AKI, acute kidney injury; LPS, lipopolysaccharide; THP-1, human monocytic cell line; TGF-β 1, transforming growth factor-beta 1; DN, diabetic nephropathy; STZ, streptozotocin; UUO, unilateral ureteral obstruction; MSCs-EVs, mesenchymal stem cells-extracellular vesicles; HP-MSCs-EVs, human placenta-derived MSCs-EVs; HUMSCs-EVs, human umbilical cord-derived MSCs-EVs; HWJMSC, the Human Wharton jelly-derived mesenchymal stromal; MVs, microvesicles; BM-MSCs, bone marrow-derived MSCs; VEGF, vascular endothelial-derived growth factor; HGF, hepatocyte growth factor; CASP, caspase; RragD, Ras related GTPase binding D; p-mTOR, phosphorylated mammalian target of rapamycin; LC3, light chain 3; CX3CL1, CX3C chemokine ligand 1; TLR-2, Toll-Like Receptor 2; NK cells, natural killer cells; Nrf2, Nuclear factor E2-related factor 2; SOD, superoxide dismutase; Keap1, Kelch-like ECH-associated protein 1; mtDNA, mitochondrial DNA; TECs, tubular epithelial cells; TNF-α, tumor necrosis factor-alpha; GRP78, glucose-regulated protein 78; CHOP, C/EBP homologous protein; ARE, antioxidant response element; HO-1, heme oxygenase-1; HGF, hepatocyte growth factor; DRP1, dynamin-related protein 1; TFAM, mitochondrial transcription factor A; OXPHOS, oxidative phosphorylation; ERK, extracellular signal-regulated kinase; α-SMA, α-smooth muscle actin; EMT, epithelial-mesenchymal transformation; PAI-1, plasminogen activator inhibitor-1; TIMP-1, Tissue inhibitor of matrix metalloprotease-1; IFN-γ, Interferon-γ; LTBP1, latent transforming growth factor beta-binding protein 1; Col1a1, collagen 1a1; P15, P15INK4b; P19, P19INK4d; CX3CL1, C-X3-C motif chemokine ligand 1; SHC1, Src homology 2 domain-containing transforming protein 1; CTGF, connective tissue growth factor; FN/Fib, fibronectin; US, ureteral stenosis; Col I, collagen I.

Studies have been performed to detect whether MSC-EVs can promote renal angiogenesis and reduce renal inflammatory cell infiltration. Zou et al. (2016b) showed that HUMSC-EVs could directly deliver human VEGF to renal tubular epithelial cells and promote angiogenesis in a model of renal tubular epithelial cell hypoxia injury and in a rat model of AKI induced by right nephrectomy and left renal ischemia for 45 min. HUMSC-EVs could also deliver relevant RNAs to renal tubular epithelial cells, up-regulate VEGF and promote angiogenesis. Zou et al. (2016a) also found, in the same rat model of renal ischemia-reperfusion injury (IRI), HUMSC-EVs reduced the expression of CX3CL1 and TLR-2 in the kidney, thereby inhibiting the up-regulation of NK cells and alleviating renal inflammation. This process might be related to the transfer of miRNAs from MSC-EVs to renal tubular epithelial cells. Zou (2014) found that in a rat model of IRI induced by left kidney ischemia for 60 min, HWJMSC-MVs injected into the tail vein could reduce infiltration of macrophages in renal tissue, apoptosis of renal tubular epithelial cells, and expression of chemokine CX3CL1 in the ischemic kidney, thus ultimately alleviating inflammation and improving RF. Du et al. (2021) found that in a rat model with ischemia-partial nephrectomy (left kidney ischemia for 45 min, 1/3 of left upper kidney resection) and in a vitro culture model of LPS-stimulated THP-1 macrophages and HWJMSC-MVs, HWJMSC-MVs could promote polarization of M2 macrophages through direct transfer of hepatocyte growth factor (HGF), thereby improving RF after ischemia. Wang et al. (2020a) pointed out that MSC-EVs could target macrophages in tissues and induce the polarization of macrophages from M1 to M2, thus playing an anti-inflammatory and immunological role and having a certain protective effect on kidney. Therefore, MSC-EVs can promote renal angiogenesis by up-regulating VEGF, reduce renal inflammatory cell infiltration by reducing CX3CL1 and TLR-2, and promote polarization of M2 macrophages by directly transmitting HGF to improve RF.

**FIGURE 2:**
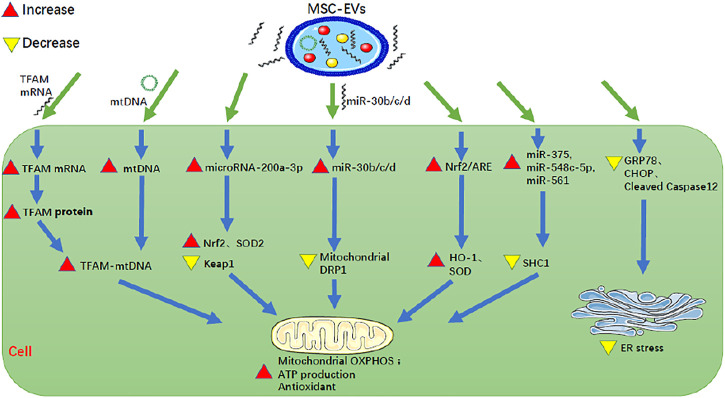
MSC-EVs can reduce mitochondrial damage of renal cells, enhance antioxidant capacity, and inhibit ER stress of renal cells. MSC-EV, mesenchymal stem cell-extracellular vesicle; TFAM, mitochondrial transcription factor A; mtDNA, mitochondrial DNA; Nrf2, nuclear factor E2-related factor 2; ARE, antioxidant response element; GRP78, glucose-regulated protein 78; CHOP, C/EBP homologous protein; SOD, superoxide dismutase; Keap1, Kelch-like ECH-associated protein 1; DRP1, dynamin-related protein 1; HO-1, heme oxygenase-1; SHC1, Src homology 2 domain-containing transforming protein 1; OXPHOS, oxidative phosphorylation; ER stress, endoplasmic reticulum stress.

2) At the cellular level, MSC-EVs can reduce mitochondrial damage and inhibit endoplasmic reticulum (ER) stress, epithelial-mesenchymal transformation (EMT), and apoptosis:

①MSC-EVs reduce mitochondrial damage of renal cells and enhance antioxidant capacity (Figure 2; Table 1):

Mitochondria produce ATP through oxidative phosphorylation (OXPHOS), to maintain the normal life activities of cells. Therefore, mitochondrial dysfunction is an important factor in tissue damage and cell aging (Zhao et al., 2021). MSC-EVs can protect mitochondria to some extent. Lindoso et al. (2014) found that in a cellular model of IRI where human kidney 2 proximal tubular epithelial cells (HK-2 PTECs) were depleted of ATP (incubation in serum-free, low-glucose medium containing 10 mM 2-deoxyglucose, and 1 mM antimycin A for 1 h), human BM-MSC-EVs reduced the expression of Src homology 2 domain-containing transforming protein 1 (SHC1) by up-regulating miR-375, miR-548C-5p, and miR-561, thereby inhibiting the excessive production of reactive oxygen species, and then inhibiting mitochondrial depolarization and alleviating mitochondrial dysfunction. Zhao et al. (2021) found that in a model of HK-2 cells with oxidative damage induced by 0.4 mM H_2_O_2_ for 48 h, the repair of oxidative damage by HUMSC-EVs occurred partly through direct transfer of mRNA for mitochondrial transcription factor A (TFAM), a primary binding and packaging protein of mitochondrial DNA (mtDNA) to recipient cells, to stabilize the TFAM-mtDNA complex, thus preventing the loss of mtDNA caused by defective OXPHOS-mediated oxidative damage. One the other hand, it was also through the direct transfer of mtDNA into the recipient cells and then into the mitochondria that mtDNA-coding genes were up-regulated to enhance mitochondrial OXPHOS and alleviate the mitochondrial dysfunction. Zhang et al. (2016) found that in a rat model with AKI induced by right nephrectomy and left renal ischemia for 45 min and in a model of hypoxia-injured NRK-52E cells, HWJMSC-EVs increased the activity of the Nrf2/antioxidant response element (ARE) in the nucleus, and then up-regulated the expression of ARE-regulated antioxidant enzymes HO-1 and SOD, thereby reducing renal tubular injury, improving renal function and effectively treating AKI. Cao et al. (2020) found that in mouse AKI models induced by ischemia-reperfusion, HP-MSC-EVs increase the expression of microRNA-200a-3p in renal tubular epithelial cells, thereby increasing the expression of the Nrf2 and SOD2, decreasing the expression of Keap1, and ultimately activating Keap1-Nrf2 signaling pathway. In this way, HP-MSC-EVs activated mitochondrial antioxidant defenses, promoted ATP production, reduced mitochondrial fragmentation, normalized mitochondrial membrane potential, and increased the copy number of mitochondrial DNA, so as to protect renal tubular epithelial cells from oxidative damage. Gu (2016) found that in a rat model with unilateral renal IRI induced by right nephrectomy and left renal ischemia for 45 min followed by reperfusion, HWJMSC-EVs could inhibit the increase of mitochondrial DRP1 expression by transmitting miR-30b/c/d to renal tubular epithelial cells, so as to restore mitochondrial dynamic balance, thus reducing apoptosis and renal injury.

To summarize, MSC-EVs can improve RF by reducing mitochondrial damage of renal cells and enhancing cellular antioxidant capacity in the following ways: 1). up-regulating miR-375, miR-548C-5p, and miR-561 to reduce SHC1, 2). direct transfer of TFAM mRNA and mtDNA into the recipient cells, 3). increasing the activity of Nrf2/ARE to up-regulate HO-1 and SOD, 4). increasing microRNA-200a-3p to increase Nrf2 and SOD2, to decrease Keap1 and to activate the Keap1-Nrf2 signaling pathway, 5). inhibiting the increase of mitochondrial DRP1 by directly transmitting miR-30b/c/d.

②MSC-EVs inhibit ER stress of renal cells (Figure 2; Table 1):

ER stress is a process in which cells activate signal pathways, such as the unfolded protein response, endoplasmic reticulum overload response and caspase-12-mediated apoptosis pathway, to cope with the accumulation of misfolded and unfolded proteins and the disruption of calcium balance in the endoplasmic reticulum. It is an important mechanism for regulating cell injury and participates in the process of RF (Liu et al., 2018). ER activation is associated with RF. MSC-EVs can inhibit ER stress of renal cells to ameliorate RF. Liu et al. (2020) found that in a mouse model of IRI, HP-MSC-EVs injected into the renal cortex exert anti-fibrotic effects in the kidney by reducing the expression of endoplasmic reticulum stress-related proteins, such as GRP78, CHOP, and cleaved caspase12, to inhibit ER stress. Therefore MSC-EVs can inhibit ER stress of renal cells to ameliorate RF by reducing GRP78, CHOP, and cleaved caspase-12.

**FIGURE 3:**
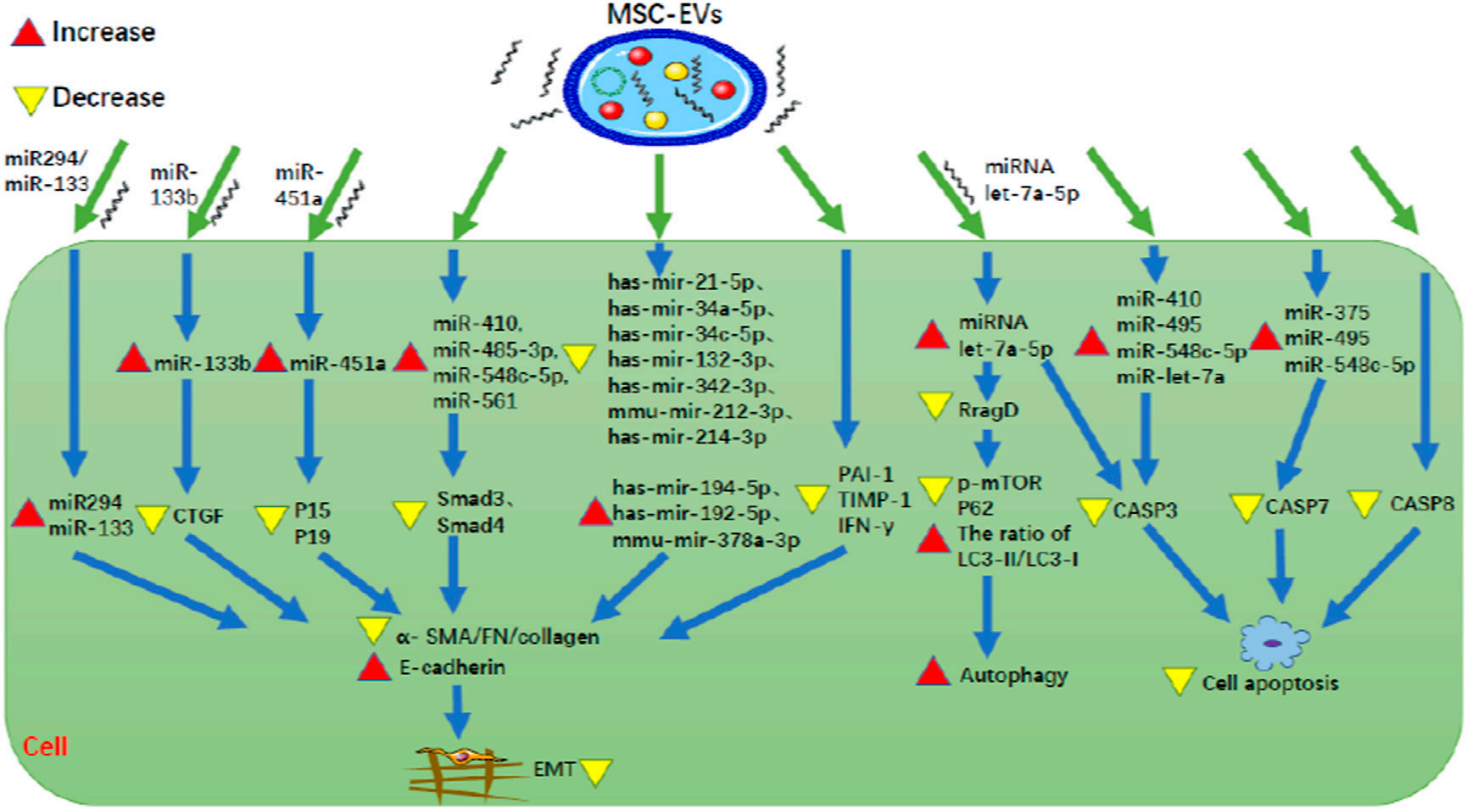
MSC-EVs can inhibit both EMT and apoptosis of renal tubular cells. MSC-EV, mesenchymal stem cell-extracellular vesicle; CTGF, connective tissue growth factor; P15, P15INK4b; P19, P19INK4d; Smad3, SMAD family member 3; Smad4, SMAD family member 4; α-SMA, α-smooth muscle actin; FN, fibronectin; PAI-1, plasminogen activator inhibitor-1; TIMP-1, tissue inhibitor of matrix metalloprotease-1; IFN-γ, interferon-γ; RragD, Ras related GTPase binding D; p-mTOR, phosphorylated mammalian target of rapamycin; LC3, light chain 3; CASP, caspase.

③MSC-EVs inhibit EMT (Figure 3; Table 1):

EMT is a process during which epithelial cells gradually transform into mesenchymal-like cells, lose their epithelial functionality and characteristics, such as like cell-cell adhesion as well as apical-basal polarity, and acquire mesenchymal characteristics that confer migratory capacity. Furthermore, numerous studies have provided evidence that EMT-derived myofibroblasts originating from tubular epithelia contribute to RF, indicating EMT plays an important role in the development of RF (Stone et al., 2016). MSC-EVs can inhibit EMT. Lindoso et al. (2014) found that in HK-2 cells following ATP depletion injury, human BM-MSC-EVs inhibit EMT and RF by up-regulating miR-410, miR-485-3p, miR-548c-5p and miR-561 so as to reduce Smad3, Smad4, and collagen. Wang et al. (2020b) found that in HK2 cells, following induction of EMT by TGF-β1 (8 ng/ml recombinant human TGF- β 1 for 48 and 72 h), rat BM-MSC-EVs could directly transfer miR-294/miR-133 to cells, thereby inhibiting the increase of α-smooth muscle actin (α-SMA) expression, the phosphorylation of SMAD 2/3 and ERK1/2, the decrease of E-cadherin expression, so as to inhibit EMT. Cao et al. (2021) found that MSC-EVs were rich in miR-133b, and miR-133b was decreased in HK-2 cells by TGF-β1 induction. In the elderly (24-month-old) mouse model with UUO, caudal vein injection of miR133b could improve renal function and interstitial fibrosis. The direct target of miR-133b is connective tissue growth factor (CTGF). Transfection of miR-133b could inhibit the down-regulation of E-cadherin and the up-regulation of α-SMA, fibronectin (FN) and collagen 3A1 (Col3A1) by directly down-regulating the expression of CTGF. Therefore, MSC-EVs can transfer miR-133b to directly inhibit CTGF, thus alleviating cellular EMT and RF. Zhong et al. (2018) found that in hyperglycemia and hyperuricemia-induced HK-2 cells and in diabetic nephropathy (DN) mice with hyperuricemia, HUMSC-MVs could prevent EMT by directly transferring miR-451a to target the 30-UTR sites of cell cycle inhibitors P15 and P19, and down-regulated the expression of P15 and P19, ultimately unblocking the cell cycle, reducing α-SMA and increasing E-cadherin. Kholia et al. (2020) found that in an AAN mouse model, MSC-EVs decreased the expression of α-SMA, TGF-β1, Col1A1, and latent-transforming growth factor beta-binding protein 1 (LTBP1) involved in the activation of TGF-β1. Additionally, MSC-EVs have been found to inhibit fibrosis, inflammation and apoptosis by down-regulating seven miRNAs (hsa-miR-21-5p, hsa-miR-34a-5p, hsa-miR-34c-5p, hsa-miR-132-3p, hsa-miR-342-3p, mmu-miR-212-3p, hsa-miR-214-3p) and up-regulating three miRNAs (hsa-miR-194-5p, hsa-miR-192-5p, mmu-miR-378a-3p). Grange et al. (2019) found that human BMMSC-EVs inhibit RF by down-regulating collagen I, TGF- β, and α-SMA in STZ-induced DN mice. MicroRNAs in EVs play an important role in the mechanism. MSC-EVs express 8 miRNAs (hsa-miR-302c-3p, hsa-let-7b-5p, hsa-miR-1243, hsa-miR-100-5p, hsa-let-7e-5p, hsa-miR-125b-5p, hsa-miR-21-5p, hsa-miR-30a-5p) that are predicted to regulate 2,154 target genes and participate in 52 common pathways, including TGF-β, EGFR, PDGFR, ARF6, mTOR, and VEGF pathways. In addition, p53-apoptosis, ATM and TNF pathways have been associated with specific MSC-EV miRNA targets. Ramírez-Bajo et al. (2020) found that in a mouse model of chronic cyclosporine nephrotoxicity (continuous peritoneal injection of 75 mg/kg cyclosporine for 4 weeks), mouse BM-MSC-EVs decreased the expression of PAI1, TIMP-1, and IFN- γ in kidney tissue, thus inhibiting EMT, reducing fibrosis, and protecting renal function. Therefore, MSC-EVs can inhibit EMT to improve RF by regulating relevant miRNAs such as miR-133b to decrease the expression of collagen I, TGF-β, and α-SMA.

④MSC-EVs inhibit apoptosis of renal tubular cells (Figure 3; Table 1):

Disfunction of apoptosis of renal tubular cells is associated with RF and studies have been conducted to determine whether MSC-EVs can inhibit this process. Lindoso et al. (2014) pointed out that caspase-3 and caspase-7 are involved in the apoptosis. In the ATP depleted HK-2 cell model, it was found that human BM-MSC-EVs decreased the expression of caspase-3 by up-regulating miR-410, miR-495, miR-548c-5p, and miR-let-7a, and decreased the expression of caspase-7 by up-regulating miR-375, miR-495, and miR-548c-5p, thus ultimately reducing cell apoptosis. Cao et al. (2020) found that in a mouse model of renal IRI, HP-MSC-EVs inhibited inflammation and apoptosis by reducing the expression of TNF-α (pro-inflammatory gene) and caspase-8 (apoptotic gene), so as to protect renal tubules and restore renal function. Zhang et al. (2020) found that in a mouse of renal IRI (ischemia for 30 min) and *in vitro* oxygen-deprived then reoxygenated HK-2 cells (HK-2 cells were exposed to low oxygen levels for 6 h, followed by 24 h of reoxygenation), miRNA let-7a-5p was induced in MSC-EVs and could play a protective role in the kidney by being transferred in extracellular vesicles. The mechanisms are as follows: 1) miRNA let-7a-5p directly targets the mRNA 3′UTR of caspase-3 mRNA, reducing the expression of caspase-3 and thus reducing renal cell apoptosis, 2) miRNA let-7a-5p directly targets the mRNA 3′UTR of RragD mRNA to inhibit the expression of RragD, thus inhibiting p-mTOR and p62, as well as increase the ratio of LC3-II/LC3-I, which ultimately down-regulates the amino acid-sensing pathway, activates autophagy, and thus inhibits cell apoptosis (Zhang et al., 2020). Therefore, MSC-EVs inhibit apoptosis of renal tubular cells to ameliorate RF by decreasing caspase-3, caspase-7, caspase-8, and RragD, some of which are downregulated by miRNAs such as miRNA let-7a-5p.

## Discussion

RF is an important pathological feature of renal aging and chronic renal failure, which can be regulated by MSCs through MSC-secreted EVs. In this review, we summarize studies demonstrating that MSC-EVs can improve RF by inhibiting inflammatory cell infiltration, promoting angiogenesis, reducing mitochondrial dysfunction, and inhibiting ER stress and EMT, as well as apoptosis. As a non-cell membrane structure, MSC-EVs not only have the advantages of low immunogenicity, easy preservation, and artificial modification, but also lack the characteristics of self-replication and ectopic differentiation. Therefore, MSC-EVs are safer than MSCs, and are expected to become one of the acellular therapies for RF.

In addition, a large number of studies have found that MSC-EVs alleviate RF mainly by transducing miRNAs to affect the stability of individual mRNAs of target cells or by transducing proteins (such as enzymes rather than structural proteins) to initiate biological reactions in recipient cells (Toh et al., 2018; Albanese et al., 2021). In this review, we show that MSC-EVs can directly transmit proteins (such as VEGF) or miRNAs (such as miR-294, miR-133, miR-133b, miR-451a, miRNA let-7a-5p, and miR-30b/c/d), or induce target cells to express related miRNA (such as miR-410, miR-495, and miR-548c-5p), thus affecting protein expression and related signal pathways, in particular the Nrf2/ARE and Keap1-Nrf2 signaling pathways, to ameliorate RF.

However, if MSC-EVs are to be applied toward clinical treatment, there are still challenges that need to be overcome and some limitations. First, MSC-EVs contain complex mixtures of components, including different kinds of RNAs and proteins, and their protective mechanisms against RF have not been thoroughly studied. So, further research is needed to find new mechanisms and specific components of EVs, so as to achieve a better understanding of the therapeutic effects of MSC-EVs on RF. Secondly, it is thought that EV mediates protection of cells by transmitting miRNA and protein, but in order for miRNAs and proteins in EVs to elicit biologically relevant activity, there are some requirements. On one hand, they must be in a biologically functional configuration. For enzymes, they can be directly assessed by enzyme activity. For miRNAs to be functional, the miRNAs in EVs must either be mature miRNAs in RNA-induced silencing complexes (RISCs) or pre-miRNAs that could be loaded into RISCs. On the other hand, the candidate protein or miRNA in a therapeutic dose must have sufficient functional activity to elicit a biological response of the target cell, which requires sufficient concentrations of protein or miRNA (Toh et al., 2018). Recently, Chevillet et al. (2014) quantitatively and stoichiometrically analyzed the miRNA content of exosomes, but found that most individual exosomes in standard preparations did not carry biologically significant numbers of miRNAs and therefore were individually unlikely to be functional as vehicles for miRNA-based communication. Albanese et al. (2021) also found that only a small fraction of EVs carry miRNAs and miRNAs are rarely delivered to target cells. However, these two study results are not enough to negate some of the previous experimental results we summarized, because their cellular sources of EVs were different. Therefore, further studies are needed to investigate whether miRNAs and proteins in EVs are biologically sufficient, and whether and to what extent EVs carry and release their miRNA and protein cargo in a paracrine manner to diverse types of recipient cells. Thirdly, MSC-EV preparation has wide variability permeating the whole process from the source of the initial cell to the production and purification of the final product. In other words, different MSC sources, culture conditions and media, as well as EV harvesting strategies may introduce significant differences in the preparation of MSC-EVs. So far, no recommended standard techniques have been established for clinical grade production of EVs. Therefore, several manufacturing and safety considerations need to be addressed and a quantifiable method of analysis for controlling the quality of MSC-EVs is also needed (Lener et al., 2015; Witwer et al., 2019; Gimona et al., 2021). Fourthly, the biological activity and treatment use of MSC-EVs might also be easily affected by storage conditions, biomaterial transport carrier, input path (intravenous administration or intraperitoneal administration; local administration or systemic administration), input timing (intermittent administration or continuous administration), and therapeutic dose. Therefore, more research should be carried out to find the best way to prolong the half-life of EVs *in vivo* and *in vitro*, and develop specific treatment schemes to obtain better therapeutic efficacy.

In conclusion, MSC-EVs can improve RF by transmitting miRNAs or proteins, some of which have been summarized in this review. However, if we want to apply MSC-EVs to the clinical treatment of RF, further research will need to be carried out to overcome the challenges mentioned above.

## Glossary

AAN: aristolochic acid nephropathy
AKI: acute kidney injury
APF: arteriosclerosis and perivascular fibrosis
ARE: antioxidant response element
α-SMA: α-smooth muscle actin
BM-MSCs: bone marrow-derived mesenchymal stem cells
BUN: blood urea nitrogen
CKD: chronic kidney disease
Col3A1: collagen 3A1
Cr: creatinine
DN: diabetic nephropathy
EMT: epithelial mesenchymal transformation
ER: endoplasmic reticulum
EVs: extracellular vesicles
FN: fibronectin
GS: glomerular sclerosis
HGF: hepatocyte growth factor
HP-MSC: human placenta derived mesenchymal stromal cell
HUMSCs: human umbilical cord-derived mesenchymal stromal cells
HWJMSC: human Wharton’s Jelly mesenchymal stromal cells
IRI: ischemia-reperfusion injury
Kim-1: kidney injury molecule-1
LTBP1: latent-transforming growth factor beta-binding protein 1
MSCs: mesenchymal stem cells
mtDNA: mitochondrial DNA
MVs: micro vesicles
OXPHOS: oxidative phosphorylation
PN: partial nephrectomy
RF: renal fibrosis
RIF: renal interstitial fibrosis
STZ: streptozotocin
TGF-β1: transformational growth factor -β1
UA: uric acid
UUO: unilateral ureteral obstruction
